# A real-time phenotyping framework using machine learning for plant stress severity rating in soybean

**DOI:** 10.1186/s13007-017-0173-7

**Published:** 2017-04-08

**Authors:** Hsiang Sing Naik, Jiaoping Zhang, Alec Lofquist, Teshale Assefa, Soumik Sarkar, David Ackerman, Arti Singh, Asheesh K. Singh, Baskar Ganapathysubramanian

**Affiliations:** 1 Department of Mechanical Engineering, Iowa State University, Ames, IA 50011 USA; 2 Department of Agronomy, Iowa State University, Ames, IA 50011 USA

**Keywords:** High-throughput phenotyping, Image analysis, Machine learning, Plant stress, Smartphone

## Abstract

**Background:**

Phenotyping is a critical component of plant research. Accurate and precise trait collection, when integrated with genetic tools, can greatly accelerate the rate of genetic gain in crop improvement. However, efficient and automatic phenotyping of traits across large populations is a challenge; which is further exacerbated by the necessity of sampling multiple environments and growing replicated trials. A promising approach is to leverage current advances in imaging technology, data analytics and machine learning to enable automated and fast phenotyping and subsequent decision support. In this context, the workflow for phenotyping (image capture → data storage and curation → trait extraction → machine learning/classification → models/apps for decision support) has to be carefully designed and efficiently executed to minimize resource usage and maximize utility. We illustrate such an end-to-end phenotyping workflow for the case of plant stress severity phenotyping in soybean, with a specific focus on the rapid and automatic assessment of iron deficiency chlorosis (IDC) severity on thousands of field plots. We showcase this analytics framework by extracting IDC features from a set of ~4500 unique canopies representing a diverse germplasm base that have different levels of IDC, and subsequently training a variety of classification models to predict plant stress severity. The best classifier is then deployed as a smartphone app for rapid and real time severity rating in the field.

**Results:**

We investigated 10 different classification approaches, with the best classifier being a hierarchical classifier with a mean per-class accuracy of ~96%. We construct a phenotypically meaningful ‘population canopy graph’, connecting the automatically extracted canopy trait features with plant stress severity rating. We incorporated this image capture → image processing → classification workflow into a smartphone app that enables automated real-time evaluation of IDC scores using digital images of the canopy.

**Conclusion:**

We expect this high-throughput framework to help increase the rate of genetic gain by providing a robust extendable framework for other abiotic and biotic stresses. We further envision this workflow embedded onto a high throughput phenotyping ground vehicle and unmanned aerial system that will allow real-time, automated stress trait detection and quantification for plant research, breeding and stress scouting applications.

**Electronic supplementary material:**

The online version of this article (doi:10.1186/s13007-017-0173-7) contains supplementary material, which is available to authorized users.

## Background

Soybean (*Glycine max* (L.) Merr.) is a huge source of revenue for the United States, with production of approximately USD 40 billion in 2014 [1]. There are various factors that affect soybean yield, such as nutrient availability, weed management, genetics, row configuration, stress (biotic and abiotic) and soil fertility [2]. Iron deficiency chlorosis (IDC) is a yield-limiting abiotic stress which affects plants that usually grow on calcareous soil with high pH. Soybean plants growing in calcareous soils (soils with free calcium carbonate and high pH) are unable to uptake iron from the soil leading to iron deficiency in plants. IDC causes reduced plant growth leading to a reduction in yield potential and quality of the crop. In the mid-west USA, IDC is one of the major problems reducing soybean yield, by as much as 20% for each visual rating point [3]. This causes an estimated economic loss of $ 260 million in 2012 alone [4]. IDC symptoms are observed at early plant growth stages on newly grown leaf tissue where chlorosis (yellowing) occurs in between the veins of the leaves, while the veins themselves remain green [5]. The extent of the problem varies depending on the cultivar, field and the year.

Soybean breeders in the US breed for genotypes with improved IDC tolerance by selecting for genes that help make the plant more iron uptake efficient [6]. Selection for desirable soybean genotype (with IDC tolerance) is done either through phenotyping in the field or in greenhouses [7], or genotyping with molecular markers linked to genes that improve IDC tolerance. More than 10 genes have been reported to be associated with improving IDC tolerance [8, 9] making genotyping approaches onerous where a breeding program may be working to select for several other traits. Phenotyping is most suitable as it allows identification of soybean genotypes that have an acceptable IDC tolerance. Furthermore, this method is cost effective and potentially requires little access to specialized labs.

Current methods for phenotypically measuring IDC are completely visual and labor-intensive. Rodriguez de Cianzio et al. [7] and Froechlich and Fehr [3] reported that visual scoring is the simplest, subjective measurement that requires relatively less labor. However, it has reduced accuracy if the evaluation is made in diverse environments and by different raters [10]. In addition, there can be intra-rater repeatability or inter-rater reliability [11] issues leading to incorrect visual scores. It also depends on the subjectivity (and its variability) of the IDC rater. Specifically, the human eye can get tired after long hours of scoring plants for various traits, which can produce large intra-rater variability in rating scores, thus resulting in diminished accuracy and reproducibility. In a breeding program, hundreds or thousands of plots are rated in a short time frame. A short time frame is crucial because one has to minimize plant stage variability, i.e., variability that is introduced if genotypes are rated over a longer time frame. *It is therefore essential to develop methods that allow for unbiased, accurate, cost effective and rapid assessment for IDC in particular, and plant biotic (e.g., diseases) and abiotic stresses in general*. There has been recent work in this regard to design, develop and deploy high efficiency methods/tools to quantify leaf surface damage [12] as well as plants response to pathogens [13]. Additionally, a number of approaches using imaging methods for phenotyping, such as fluorescence and spectroscopic imaging have been successful for stress-based phenotyping [14], high throughput machine vision systems that use image analysis for phenotyping *Arabidopsis thaliana* seedlings [15] and barley [16], hyperspectral imaging for drought stress identification in cereal [17], and a combination of digital and thermal imaging for detecting regions in spinach canopies that respond to soil moisture deficit [18] which have proven to be successful. However, a simple, user friendly framework is unavailable for the public to phenotype for IDC in soybean plants. The availability of a simple modular approach could potentially be generalized for phenotyping of multiple stresses.

Motivated by these reasons, we developed a simple framework (image capture → data storage and curation → trait extraction → machine learning/classification → models/smartphone apps for decision support) that extracts features that are known to quantify the extent of IDC (amount of yellowing, amount of browning) from digital images. To determine a relationship between these features and their respective ratings, we evaluated a host of machine learning techniques, further elaborated in the latter stages of this paper, to perform supervised classification. Subsequently, using information obtained from these classifiers, a physically meaningful population canopy graph (PCG) connecting the features with the visual IDC rating was constructed for a diverse soybean germplasm. This complete framework, which is based on fast feature extraction and classification, can then be used as a high throughput phenotyping (HTP) system for real time classification of IDC. We enable real time phenotyping by implementing the software framework as a GUI-based, user-friendly software that is also deployed on smartphones. This step successfully abstracts the end-user from the mathematical intricacies involved, thus enabling widespread use. We showcase this software framework by extracting IDC features (amount of yellowing, amount of browning) from a set of 4366 plants that have different IDC resistances.

We envision our classifier based framework as a modular, extensible and accurate phenotyping platform for plant researchers including breeders and biologists.

## Methods

### Genetic material and field phenotyping

A total of 478 soybean genotypes, including 3 maturity checks and 475 soybean plant introduction (PI) lines acquired from the USDA soybean germplasm collection, were planted in the Bruner farm in Ames, IA, 2015, where soybean IDC was present in previous years. This set of PI lines exhibits a wide diversity in leaf and canopy shape [19]. The design for this field experiment follows a randomized complete block design, with a total of four replications. Each PI line was planted once per replication, while the IDC checks (two) and maturity checks (three) were repeated at regular intervals in the field with four plots per replication. The plants were planted in 0.31 m length hill plot of five seeds per plot. At two soybean growth stages [20]: the second to third trifoliate (V2–V3) and fifth to sixth trifoliate (V5–V6) leaf stages, the soil pH was tested in the Soil and Plant Analysis Laboratory, Iowa State University. At each stage, eight soil samples were randomly collected from each replication and were mixed as one test sample. The soil pH values ranged at 7.80–7.95 and 7.75–7.85 at V2–V3 and V5–V6 growth stages, respectively. Field visual ratings (FVR) of IDC severity by experts were collected at V2–V3 and V5–V6 growth stages, as well as two weeks after the V5–V6 stage to obtain soybean canopies with a variety of IDC expression. FVR was done on a scale of 1–5 described by Lin et al. [21], where 1 indicates no chlorosis and plants were normal green; 2 indicates plants with modest yellowing of upper leaves; 3 indicates plants with interveinal chlorosis in the upper leaves but no stunting growth; 4 indicates plants are showing interveinal chlorosis with stunting growth; and 5 indicates plants show severe chlorosis plus stunted growth and necrosis in the new youngest leaves and growing points.

### Image acquisition

We utilized a Canon EOS REBEL T5i camera for image acquisition. Images were stored in the native RAW format. Substantial effort was put in to develop a standard imaging protocol (SIP) (Additional file 1) to ensure imaging consistency and quality. The flash function was kept off and an umbrella was always used to shade the area under the camera view in order to minimize illumination discrepancies between images. A light/color calibration protocol was also followed. An image of a color calibration chart (X-Rite ColorChecker Color Rendition Chart) was taken at the beginning of imaging operations, and every 20 min thereafter or whenever light condition changes (cloud cover, etc.). When taking pictures, the whole canopy was fit in the field of view of the camera. Weed control was practiced consistent with research plots and commercial farms; however, due to the small size of the field weed removal was done manually. Weeds in the view of camera were removed for enhanced efficiency of subsequent image processing. Images were taken across several days (at several times of the day) under various illumination conditions. Finally, the imaging protocol was chosen so that the imaging window and the camera resolution resulted in images with at least 6 pixels/mm, ensuring that the approach is transferable to other cameras that use an appropriate imaging window to get this resolution.

### Dataset description

A total of 5916 RGB (493 plots including PI accessions and checks × 4 replications × 3 time points) images were acquired, along with subsequent FVR. Each time point consists of four repetitions for a total of 1972 (493 × 4) images, with 493 images per repetition. Image acquisition at each of these time points was vital to obtain a large variety of IDC symptoms, as IDC symptoms progress in time. The idea was to develop a dataset with similar number of observations per IDC rating. This was, however, not possible simply due to the fact that a large fraction of plants remained healthy (FVR = 1) throughout the image acquisition period. Following image acquisition, for quality control, each image was inspected visually, and those that did not adhere to the Standard Imaging Protocol (SIP) were removed, which resulted in 4366 images in the remaining image set.

### Preprocessing and feature extraction

#### Preprocessing

##### White balance and color calibration

As the appearance of color is affected by lighting conditions, using a calibration chart enables color correction to be applied to ensure that colors are uniform throughout all the plant canopy images collected. We primarily used the grey squares to identify the white balance, while the green, brown and yellow squares were used to calibrate the hue values of green, brown and yellow. Hue is defined as the color or tint of an object. Hue quantifies color in terms of angle around a circle (or more precisely around a color hexagon) with values ranging from 0° to 359° [22]. The red color axis is usually set as 0°. The hue of brown ranges from 21° to 50°, whereas yellow hue ranges from 51° to 80° [23]. Calibration is done by identifying how much the hue value of the green, brown and yellow squares on the color calibration chart has drifted from the defined hue values. This drift correction is then applied to the canopy images. This preprocessing resulted in an analysis pipeline that was robust to changes in illumination.

##### Segmentation

Each image was converted from native Red, Green, Blue (RGB) format to HSV (Hue, Saturation, Value) format [22] to efficiently perform background removal, leaving only the plant canopy (foreground). The background of an image (soil, debris) contains more gray pixels compared to the foreground (plant), and lacks green and yellow hue values; therefore, most of the background was removed by excluding pixels that had saturation value below a predefined threshold and hue values outside of a predefined range. The saturation threshold value was obtained by identifying the saturation values of the background in 148 diverse images. The hue range was simply obtained from the hue color wheel, removing pixels that were neither green nor brown. This combined thresholding based on incorporating hue thresholding with saturation thresholding ensured a reliable and robust segmentation process.

##### Noise and outlier removal

**Fig. 1:**
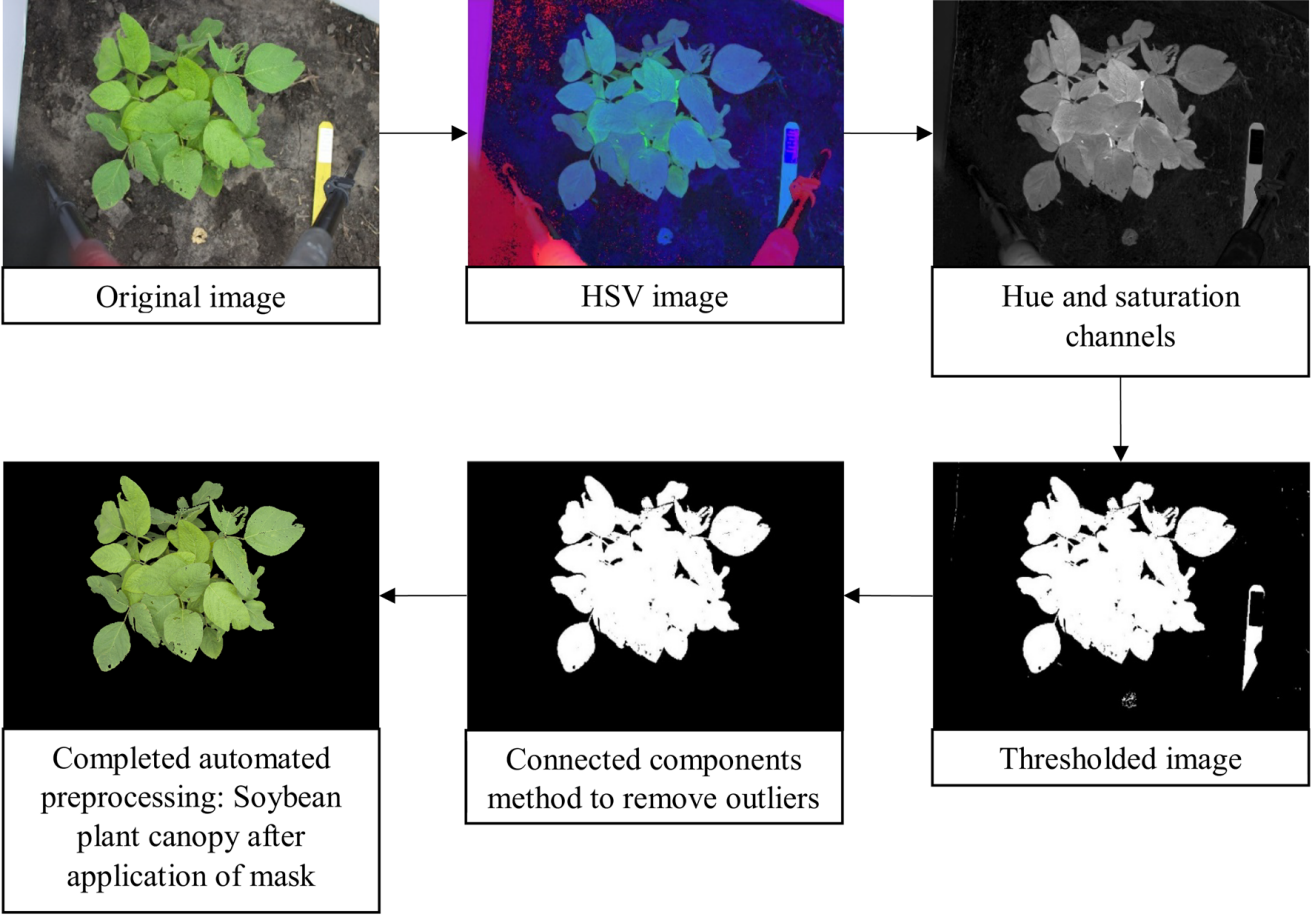
Image preprocessing sequence from original image of canopy to completed automated pre-processed field soybean canopies

Once segmentation was done, the connected components method [24] was used on the processed image to remove spurious outliers and noise from the image, (for example, plant debris on soil). This was accomplished by identifying clusters of pixels which are connected to one another, labelling them, and identifying the largest connected component. Since the imaging protocol was designed to ensure that the plant was centered in the imaging window and in the foreground, it follows that the largest connected component is invariably the plant. Cleaning was done by removing any other connected components that contain fewer pixels than the largest connected component. Then, a mask of the isolated plant was applied onto the original RGB image in order to display the isolated plant in color. No significant pixel loss was observed which is common in other thresholding methods [25]. The use of the connected components approach to isolate plants from background is extremely fast and accurate. In conjunction with a SIP, using connected components for preprocessing is very promising, especially for near real time phenotyping applications. The preprocessing sequence is illustrated in Fig. 1.

#### Feature extraction from expert elicitation

**Fig. 2:**
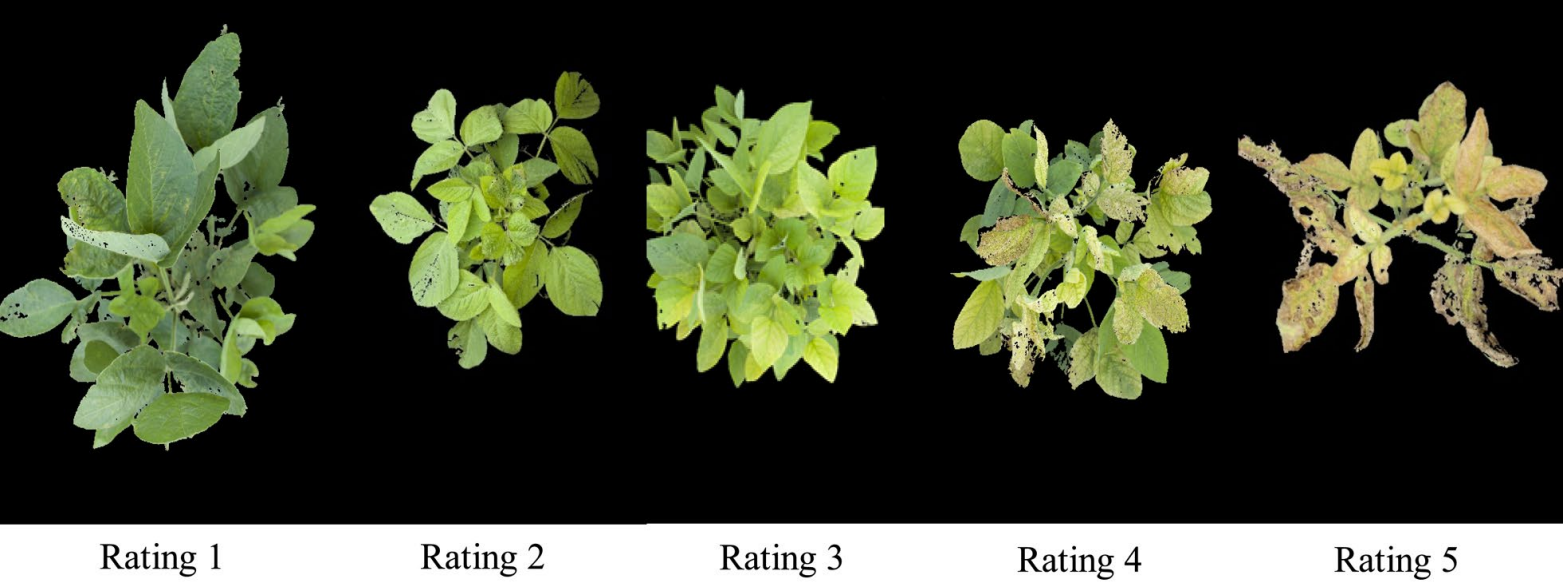
Iron deficiency chlorosis severity description using a field visual rating scale of *1–5*

**Fig. 3:**
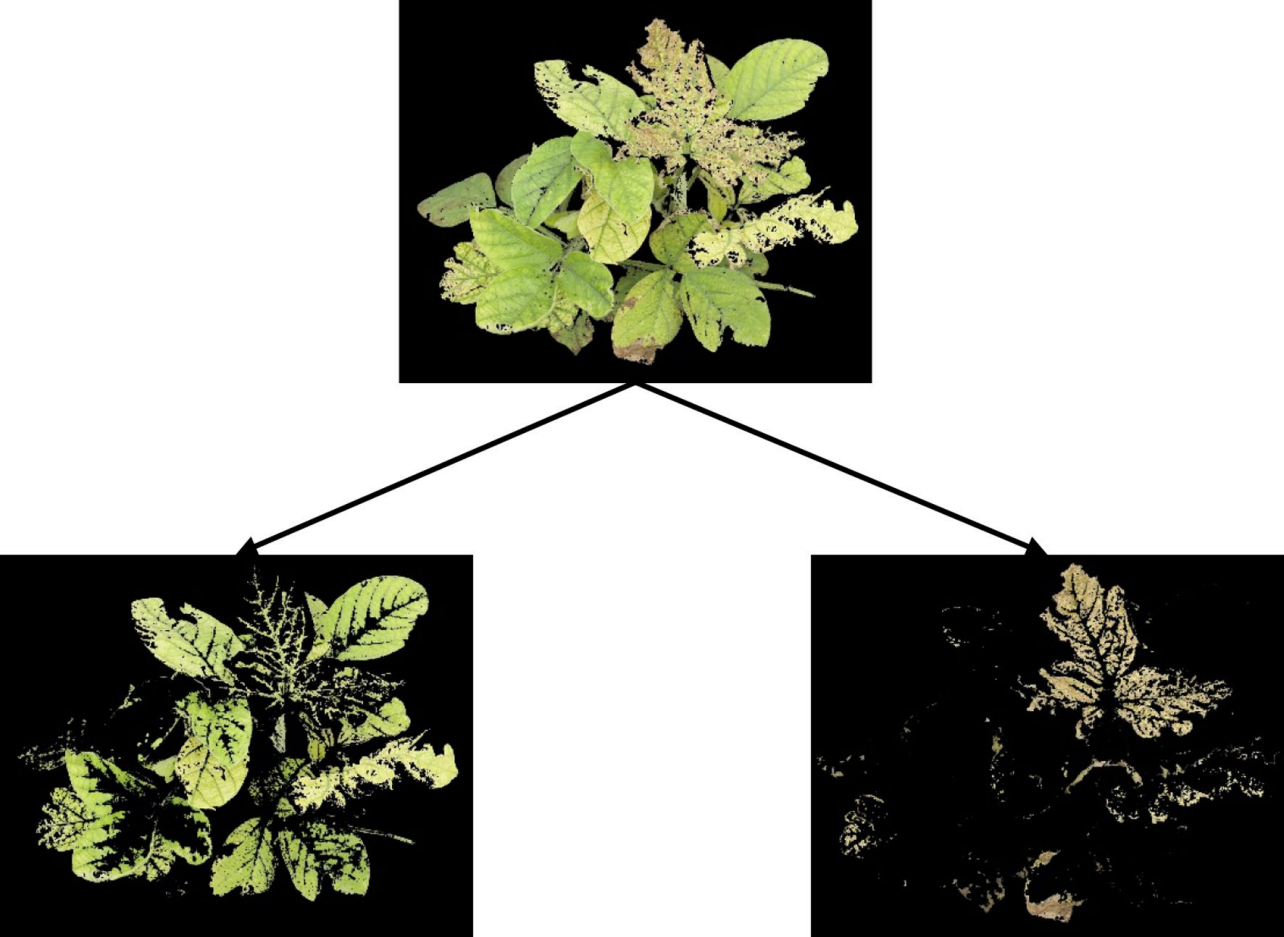
Feature extraction from plant canopies (*top image*) for iron deficiency chlorosis. The *bottom left figure* represents those regions in the canopy that are *yellow* in color, and the *bottom right figure* represents those regions in the canopy that are *brown* in color. The percentage spread of *yellow* and *brown* color are then taken as the two features

Field visual ratings are assigned based on the extent of chlorosis (yellowing) and necrosis (browning) expressed on the canopy, as described earlier and illustrated in Fig. 2. Elicitation from domain knowledge experts (i.e., raters) suggested that color signatures (green to yellow to brown), specifically extent of (dis)coloration (chlorosis → yellowing, and necrosis → browning) were viable predictors to quantify IDC expression. Each pixel of the processed image belonging to the canopy was identified as either green, yellow, or brown through evaluating their respective hue values to identify which hue ranges they belong to, and the extent of discoloration from green was represented in the form of the percentage of canopy area that experience these visual changes (Y and B%), as seen in Fig. 3.

1 $$\frac{{Area_{yellow} }}{{Area_{total} }} \times 100\% = Percentage_{yellow} , \quad \frac{{Area_{brown} }}{{Area_{total} }} \times 100\% = Percentage_{brown}$$

This expert elicitation informed processes resulted in each image being represented by a quantitative measure of yellowing (Y%) and browning (B%), as shown in Eq. 1.

### Classification

In order to map these quantitative variables to the visually rated IDC ratings, we utilize several state of the art machine learning algorithms to construct classification models. The field visual rating served as the categorical output variable (classes) while the inputs were the 2-tuple (Y, B%). The classification models are then eventually used to generate IDC ratings given different input variables.

The total dataset consisted of 4366 images following quality control as detailed in the “Dataset description” section. The images were sorted into 5 groups which correspond to their respective FVR, with majority of the observations falling into group 1 (FVR = 1). The remaining groups (FVR = 2/3/4/5) meanwhile contained a balanced distribution of observations amongst themselves.

Due to the imbalanced nature of the dataset with a preponderance of images belonging to FVR 1, two variations of the dataset were used to develop classification models: (a) Using observations from time point 2 and (b) for a combination of time point 1, 2, and 3. Time point 2 served as a standalone dataset due to the fact that it has the largest distribution of observations containing each of the FVRs. We utilized several classification algorithms, namely classification trees (CT), random forests (RF), Naïve Bayes (NB), linear discriminant analysis (LDA), quadratic discriminant analysis (QDA), multi-class support vector machines (SVM), k-nearest neighbors (KNN), and Gaussian mixture models (GMM). Building upon the results, we subsequently utilized the concept of hierarchical classification to develop two additional models using a combination of LDA and SVM algorithms.

The dataset was randomly sampled into two subsets in a 75–25% ratio. The larger subset (75%) served as the training set, while the remaining subset served as the testing dataset (25%). We additionally evaluated the performance of the classifier across additional datasets. One dataset consisted of images from completely different genetic material. Additionally, we repeated the field experiment in 2016 and used the trained classifier on images from this experiment [26]. The training dataset is used to train the classifier, by learning a mapping of the Y and B% with their expected IDC ratings. Subsequently, the testing dataset is used to estimate the performance of the classification model, by applying it on the testing dataset to classify each observation. The performance of the classifier can be interpreted from the confusion matrix (Table 1). The diagonals on a confusion matrix show the number of observations where, the predicted rating is equal to the actual rating, whereas the off-diagonal elements are observations that have been misclassified.

**Table 1:** Confusion matrix

	Predicted positive (class 1)	Predicted negative (class 2)
Actual positive (class 1)	True positive (TP)	False negative (FN)
Actual negative (class 2)	False positive (FP)	True negative (TN)

Three measures of accuracy of the classifier are reported from the confusion matrix

 An example confusion matrix for a *binary* classification problem is shown below:

- *Accuracy* which quantifies the fraction of the training dataset that is correctly predicted.
  2 $$Accuracy = \frac{TP + TN}{TP + TN + FP + FN} \times 100$$
- *Per*-*class accuracy* is a more refined metric which calculates how the classifier performs for each of the classes. This is useful when the instances in each class vary a lot, i.e., when the classes are *imbalanced (as is the case in this work)*, since accuracy is usually overestimated due to the impact of the class with the most instances dominating the accuracy statistic.
  3 $$Per\;class\;accuracy = \frac{{i{ - }th\;observation\;of\;row\;i}}{Sum\;of\;observations\;of\;row\;i},\quad i=1,\ldots,n,$$
  where *n* number of classes, *row* row on the confusion matrix
- *Mean per*-*class accuracy (MPCA)* is the mean per-class accuracy over these classes.
  4 $$Mean\;Per\;Class\;Accuracy = \frac{1}{n}\mathop \sum \limits_{i = 1}^{n} Per\;class\;accuracy$$

In addition, we compute the misclassification costs in order to quantify the cost of the misclassification errors—i.e., if an observation in rating 1 were to be classified as rating 5, it would have a higher misclassification cost than if it were to be classified as rating 2. Essentially, calculating the misclassification cost enables us to know, if errors are made, how bad the errors are. To do so, we defined a misclassification cost matrix, as detailed in Table 2. The off-diagonals of the matrix are the misclassification cost for each of the ratings, which are finite, real values [27]. For example, if the actual rating of an observation is rating 1, the error of misclassifying the observation to rating 5 is 4 times as costly as misclassifying the observation to rating 2, and so on. Then, misclassification cost is computed using Eq. 5.

5 $$cost = \frac{1}{N}\mathop \sum \limits_{i} \mathop \sum \limits_{j} CM_{ij} * w_{ij} ,$$

*CM*_*ij*_ confusion matrix, *w*_*ij*_ cost matrix, *N* number of observations

**Table 2:** Cost matrix, w_ij_

	Predicted ratings				
Actual ratings	0	1	2	3	4
	1	0	1	2	3
	2	1	0	1	2
	3	2	1	0	1
	4	3	2	1	0

Next, we employ cross-validation to estimate the average generalization error for each classifier. Cross-validation essentially is a method of assessing the accuracy and validity of a statistical model for generalization on future datasets. From a generalizability standpoint, the absolute accuracy of a classifier is less important as it could be subject to bias and overfitting. Hence, cross-validation is a method of performance estimation based on the variance. The ideal estimation method would have low bias and low variance [28]. We used k-fold cross-validation, with k = 10 which is a good compromise between variance and bias [28]. K-fold CV was repeated 10 times to compute the mean cross-validation misclassification error for each model. While accuracy and MPCA detail the performance of a classifier on essentially the same dataset, mean cross-validation misclassification error provides information on how well the classifier performs on other datasets.

#### A brief description of classification algorithms deployed

We briefly describe each of the classification algorithms [29]. We refer the interested reader to a more detailed description of these methods in [30–32].

##### Decision trees

It is based on the construction of predictive models with a tree-like structure that correlates observations to their corresponding categories such as classes (for classification) and rewards (for decision-making problems). These observations are sorted down the tree from the root to a leaf node, which in turn classifies the observation. Decision trees [33] perform well on lower dimensional classification problems, but tend to falter when the dimension of the classes increases.

##### Random forests

An ensemble method employed to regularize the greedy, heuristics nature of the decision tree training which sometimes causes overfitting. This method [34] combines results and structures from a number of trees prior to coming to a conclusion. Multiple trees are grown from random sampling of the data. Nodes and branch choices of a tree are also determined through a non-deterministic manner. These models are more robust to uncertainties.

##### Naïve Bayes

A supervised classification technique for constructing classifiers of a probabilistic graphical model. It is based on the assumption that each feature is independent of each other. Naïve Bayes [35] have been used in a variety of fields, and is a popular method for text categorization.

##### Linear discriminant analysis (LDA)

A linear classification technique [36] based on the idea of Fisher’s Metric, with an aim to maximize between class variance, while minimizing within-class variance. This allows the linear combination of features to improve separability among two or more classes. This requires an assumption of equal variance–covariance matrices of the classes.

##### Quadratic discriminant analysis (QDA)

A modification of linear discriminant analysis, except a covariance matrix must be estimated for each class. This allows overcoming the problem where the variance–covariance differs substantially [36], where LDA will not perform well.

##### Support vector machine (SVM)

The most popular among supervised, discriminative kernel-based methods for classification. SVM [37] uses kernel functions to project data into a higher dimensional space in order to separate data from different classes which cannot be linearly separated. A hyperplane is constructed to determine the bounds in which each class is separated, to maximize class separability.

##### K-nearest neighbors (KNN)

A non-parametric classification method [38]. This algorithm assigns the same class label to data samples as its k nearest neighbors based on a similarity metric defined on the feature space, where k is an integer. This nonlinear algorithm works reasonably well for multi-class classification problems.

##### Gaussian mixture model (GMM)

A generative, unsupervised data model that aims to identify a set of Gaussian distributions mixtures which best describe the data. GMM [39] is a probabilistic technique where every data example is expressed as a sample of the distribution which is a weighted sum of k Gaussian distribution. Once this model is created, a Bayes classifier is applied in attempt to solve classification problems.

#### Hierarchical classification

We subsequently pursued a hierarchical classification strategy that is motivated by expert elicitation of information about IDC susceptibility. Hierarchical classification is known to work well on datasets with a larger number of classes but with fewer observations. The IDC data set fell into this category. Also, the task of designing the hierarchy in this classification strategy enables the inclusion of expert knowledge. Here, the hierarchical structure is predefined, based on insight and existing knowledge of class hierarchies, which then contributes to improving classification accuracy.

In this case, the hierarchies were identified based on the susceptibility of the genotypes to IDC. Specifically, rating 1 and 2 are usually taken together as low susceptibility genotypes, while rating 4 and 5 are taken together as high susceptibility genotypes. We thus designed a two-step classification strategy: In Step A, a classifier is learnt that can separate the data into low, medium and high susceptibility groups. Step B then further classifies these groups into rating 1 or 2 (for the low susceptibility group), and rating 4 or 5 (for the high susceptibility group).

**Fig. 4:**
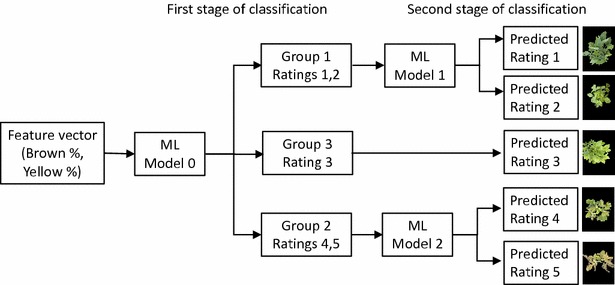
Hierarchical classifier workflow

For Step A, we deploy both LDA and multi-class SVMs. The learnt classifier is called Model 0, and seeks to classify the dataset into three groups (low, medium and high susceptibility) based on their yellow and brown percentage. For Step B, we deploy Support Vector Machine as the classification is binary. Figure 4 displays a flowchart of this hierarchical classifier.

## Results and discussion

A number of classification algorithms were capable of achieving high mean per class accuracy, more than 90%, for classification on the time point 2 data set. Hierarchical models performed relatively well, with a mean per class accuracy at 95.9%. More importantly, when the classifier made incorrect predictions, the results were predominantly within the same susceptibility class—i.e., an error in rating 1 typically falls to rating 2, and not into rating 5 etc. This is illustrated in the misclassification cost metric for each classifier, as detailed in Eq. 5. The best performing classifier, classification trees, were able to correctly predict new observations 100% of the time.

When data from all time points were used to train and test a classifier, the hierarchical model performed the best, with 91% accuracy. Other classifiers fell short of the 90% mark. The decrease in accuracy was expected simply because combining all three time points caused the data set to be more imbalanced that before.

While being able to have high classification accuracy is important, the capability of a classifier to produce an interpretable PCG was extremely vital. This is quantified by the *interpretability* of the PCG, and is further discussed in the “PCG” and “Model selection” sections.

The results of each of the classification models are displayed in Tables 3 and 4. Table 3 consists of the results from a classification model developed using a sub-set of the IDC data (which consists of 3 time points). Instead of developing a model using 3 time points, this model was developed using data from time point 2 as it has the largest distribution of observations containing each of the FVRs. Table 4 consists of results from a model developed using the data spanning across all 3 time points (the whole dataset).

**Table 3:** Results for machine learning algorithm model accuracies developed using a sub-set of iron deficiency chlorosis data on a diverse set of soybean accessions

Algorithm	Accuracy	MPCA^a^	Cross validated MPCA	Interpretability	Cost metric
CT	100.0	100.0	96.0	Medium	0.0000
KNN	99.7	96.7	95.0	Low	0.0031
RF	99.7	96.0	85.0	Low	0.0031
Hierarchy^b^	99.4	95.9	79.8	High	0.0062
QDA	99.4	92.0	98.9	Medium	0.0620
Hierarchy^c^	98.5	86.6	70.8	High	0.0155
GMMB	99.1	82.0	87.0	Medium	0.0093
NB	99.1	82.0	93.8	Medium	0.0093
LDA	98.8	79.3	84.3	High	0.0124
SVM	93.8	39.8	50.0	Low	0.1084

^a^Mean per class accuracy

^b^SVM and SVM

^c^LDA and SVM

**Table 4:** Results for machine learning algorithm model accuracies developed using the complete set of iron deficiency chlorosis data on a diverse set of soybean accessions

Algorithm	Accuracy	MPCA^a^	Cross validated MPCA	Interpretability	Cost metric
CT	99.7	91.7	78.4	Low	0.0027
Hierarchy^b^	99.2	90.7	79.2	High	0.0082
Hierarchy^c^	98.3	84.0	79.0	High	0.0201
QDA	98.5	83.2	77.9	Medium	0.0201
NB	98.4	79.0	78.5	Medium	0.0284
KNN	99.5	75.8	84.3	Low	0.0073
RF	99.1	75.0	81.1	Low	0.0092
GMMB	99.4	74.2	82.7	Low	0.0064
LDA	98.5	71.7	76.9	High	0.0156
SVM	97.3	45.8	45.3	Low	0.0458

^a^Mean per class accuracy

^b^SVM: using SVM for both classifiers

^c^LDA and SVM

### Population canopy graph

**Fig. 5:**
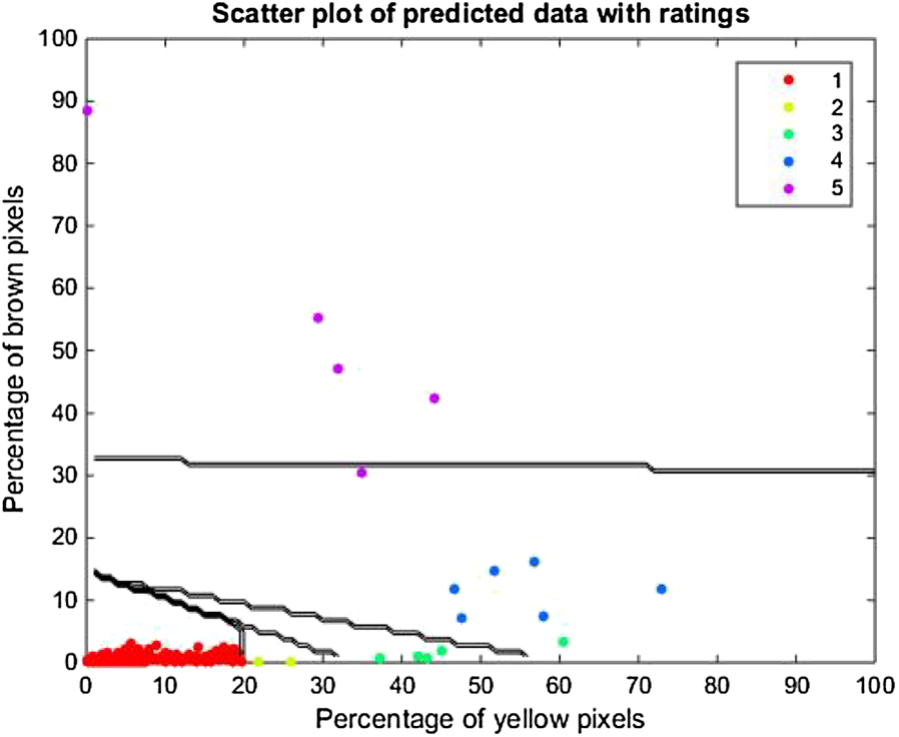
Population canopy graph of predicted data using a testing set with images and visual rating for IDC in soybean

It was interesting to note that the learnt classifier revealed insightful phenotypic intuition. Specifically, we queried the classifier to predict ratings for a uniform sampling of the Y and B% range. This information is used to construct a 2D plot that depicts decision boundaries that separate various IDC classes (as a function of Y and B%), which we refer to as a population canopy graph (PCG). This graph, shown in Fig. 5 which displays the PCG output from Hierarchy^2^ classification results on the test set, correlates very well with expert intuition. Expert intuition suggests that ratings 1–3 exhibit low brown values (corresponding to minimal to no necrosis), which is clearly seen in the PCG in Fig. 5. Similarly, beyond a certain stage of necrosis, a plant is automatically rated as 5 irrespective of the amount of chlorosis. This trend is also exhibited by the nearly horizontal line marking the Rating 5 class in Fig. 5. Finally, the linear boundaries that allow a graceful transition from rating 1 through to 2 and 3, which is similar to how experts rate the transition of chlorosis.

### Model selection

Several of the trained models exhibit good accuracy. We choose one of them as our best model based on a combination of a set of two objective measures and one subjective measure. The ideal model would have high MPCA and cross-validated MPCA as it illustrates the capability of the model to predict the IDC ratings of soybean through features extracted from images. We use MPCA instead of just accuracy due to the imbalanced nature of the dataset, as accuracy alone gives a distorted picture as the class with more examples will dominate the statistic. These two constitute the set of objective measures. Our subjective measure is based on a notion of interpretability—which we define as the ability of the end-user (plant researchers, breeders, and/or farmer) to interpret the PCG created and link it to the visible rating characteristics that are currently used. Specifically, we check to see if the shape of the decision boundaries produced by the model makes physical sense—that the decision boundaries correlated with the physical aspect of IDC, e.g.: plants with IDC rating 4 and above display significantly more browning compared to ratings 3 and below. Interpretability was scored either ‘Low’, ‘Medium’, or ‘High’; ‘Low’ for models that did not correlate with expert intuition (e.g.: individual islands, quadratic boundaries that appear to be biased), ‘Medium’ for models that partially correlates with expert intuition, and ‘High’ for models that correlated well with expert intuition. The hierarchical model Hierarchy^2^ had the best trade-offs amongst these criterions, as shown in Tables 3 and 4, and was chosen as the best model.

## Smartphone and PC software

**Fig. 6:**
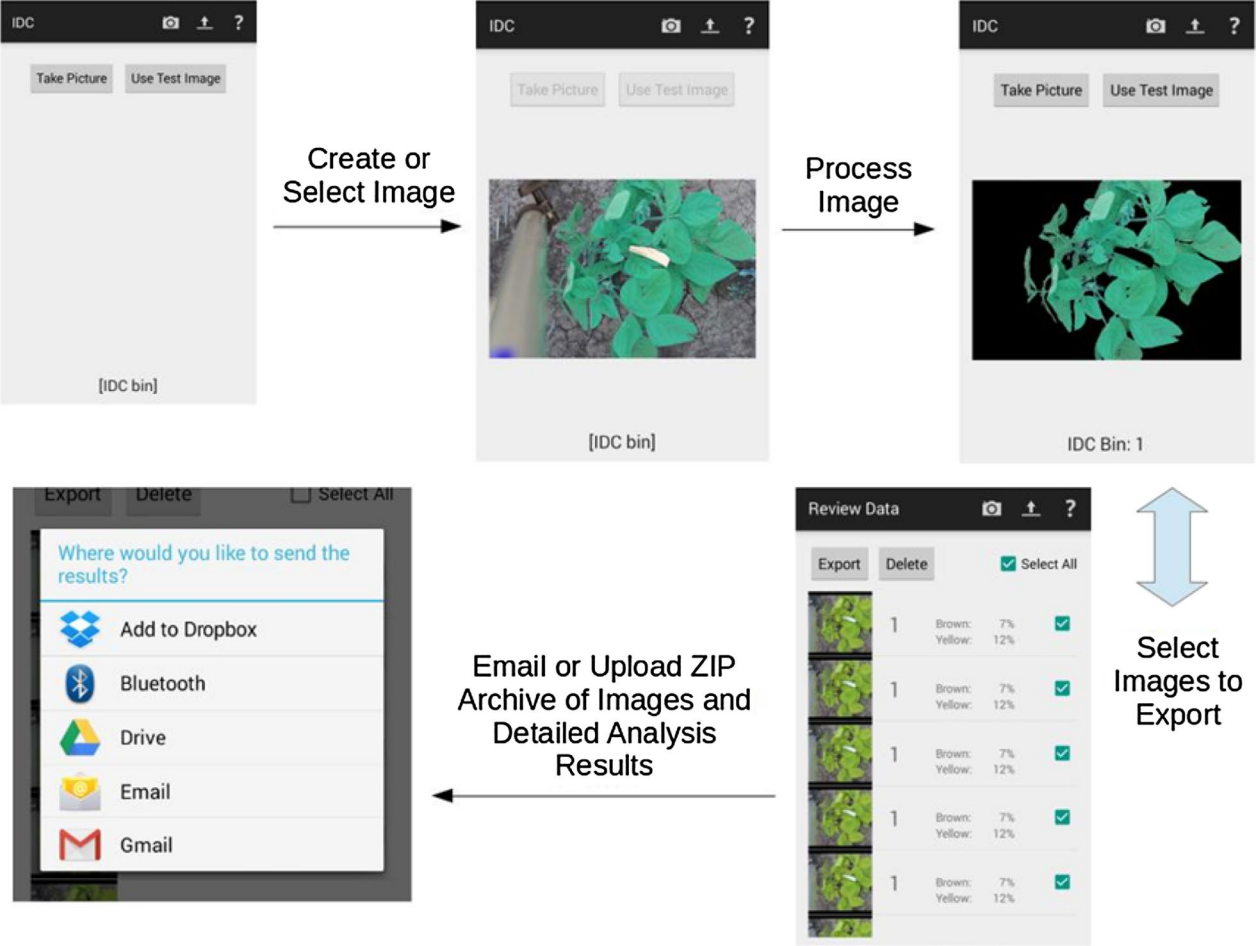
Smartphone app flowchart demonstrating the integration of pre-processing, machine learning enabled classification and iron deficiency chlorosis visual rating in real time

To enable high throughput phenotyping using the developed classifier, we embed the preprocessing stage as well as the classifier into an easy to use GUI that is deployable as a smartphone app. This app is supported on all Android-based devices, such as tablets and smartphones and has the full functionality of the desktop-based version. The Android-based app allows users to take pictures with their devices and extract the IDC rating in real time. This allows for portability and instant acquisition of data. Figure 6 shows a flowchart of illustrating the app. When the app is launched, the user has a choice between taking a new picture, and analyzing a picture already contained in the device. The picture should be taken in the native RAW format (usually in the.dng format), and not using standard JPEG formats which use lossy compression that may cause color changes. Once a picture has been selected, it is processed and the IDC score evaluated and displayed on the screen. The user can export single or multiple images in tabular form through various methods, such as Dropbox, Bluetooth, Google Drive, and through email. This app allows untrained personnel and/or unmanned ground vehicles to extract and transmit IDC ratings without the need for a trained plant researcher/phenotyper looking at every plant. This is a tremendous enabler in terms of dramatically increasing the number of plants that can be accessed. In addition to the smartphone based app, a desktop based GUI will also be released to enable batch processing of a large number of images. This allows offline (or off site) analysis of images that are either captured manually or in an automated fashion.

## Conclusion

We designed, developed and deployed an end-to-end integrated phenotyping work-flow that enables fast, accurate and efficient plant stress phenotyping. We show how image processing and machine learning can be deployed to construct classifiers that can automatically evaluate stress severity from image data. We emphasize that expert knowledge is crucial in designing appropriate classifiers. This is clearly seen in the markedly superior performance of the hierarchical classifier over single stage classifiers. The classifier is additionally used to produce a phenotypically meaningful population canopy graph. Subsequently, we deploy the developed classifier onto smartphones that serves as a high-throughput framework that can be utilized cross-platform for evaluating IDC ratings of soybean using only digital images. It is clear that image based analysis is more reliable and consistent than visual scoring as it removes the human error aspect involved in visual rating when repeated IDC measurements are needed at different growth stages. We compared the computed IDC ratings with provided visual scores from domain experts, and observed a close similarity, supporting accurate measurements and the accuracy of this HTP framework. We envision that such systems will help the plant researchers and breeders increase the efficiency and accuracy of selecting genotypes compared to visual scoring to enable fast phenotyping and reduce researcher bias. It is also relatively low cost and has the potential to speed up and improve crop development. The newly developed software framework is being embedded onto a high throughput phenotyping ground vehicle and unmanned aerial system (UAS) that will allow real-time, automated stress trait detection and quantification for plant breeding and stress scouting applications. This framework is also currently under further development by our group for numerous biotic stresses in soybean.

## Additional file

**Additional file 1.** Standard Imaging Protocol.docx. Standard Imaging Protocol (SIP) used in the collection of images.
