# Supplementary material for: A real-time phenotyping framework using machine learning for plant stress severity rating in soybean
Source: Plant Methods. 2017 Apr 8;13:23. doi: 10.1186/s13007-017-0173-7 (PMC5385078; doi:10.1186/s13007-017-0173-7)
Supplement: Supplementary file 1 — Additional file 1. Standard Imaging Protocol.docx. Standard Imaging Protocol (SIP) used in the collection of images. [file 13007_2017_173_MOESM1_ESM.docx]

Additional File 1: Standard Imaging Protocol (SIP)

1. Ensure that the lighting is consistent in all the images.
2. Always take a picture of the X-Rite Color Checker Color Rendition Chart first.
   1. Ensure that the lighting does not change after taking a picture of the chart.
   2. Should the lighting change, make a note of which image does the lighting change, and take a picture of the chart again.
3. Ensure that there are no weeds/other plants/large objects (shoes, paper etc.) in the image.
4. Ensure that only one plant is in the image.
5. If taking pictures of greenhouse plants, ensure that the background of the image is one flat color (Black preferred). Use a black cloth to cover the background.
6. Take diseased portion of plant/portions which show the most signs of symptoms in image.
7. Ensure that light is not reflected by the leaves (leaves appear white).
   1. Try not to use flash. If using a flash is a must, use a diffuser on the flash to diffuse light.
